# Cardiac Magnetic Resonance Imaging of COVID-19-Associated Cardiac Sequelae: A Systematic Review

**DOI:** 10.31083/j.rcm2312389

**Published:** 2022-11-30

**Authors:** Montek S Boparai, Benjamin Musheyev, Umair Khan, Tejaswi Koduru, Jared Hinson, Hal A Skopicki, Tim Q Duong

**Affiliations:** ^1^Department of Radiology, Montefiore Medical Center and Albert Einstein College of Medicine, Bronx, NY 10467, USA; ^2^Renaissance School of Medicine at Stony Brook University, Stony Brook, NY 11794, USA; ^3^Department of Medicine, Division of Cardiology, Renaissance School of Medicine at Stony Brook University, Stony Brook, NY 11794, USA

**Keywords:** MRI, SARS-CoV-2, myocarditis, post-acute COVID-19 symptoms, troponin

## Abstract

**Background:**

Many COVID-19 survivors experience persistent COVID-19 related cardiac abnormalities weeks to months after recovery from acute SARS-CoV-2 infection. Non-invasive cardiac magnetic resonance (CMR) imaging is an important tool of choice for clinical diagnosis of cardiac dysfunctions. In this systematic review, we analyzed the CMR findings and biomarkers of COVID-19 related cardiac sequela after SARS-CoV-2 infection.

**Methods:**

Following the Preferred Reporting Items for Systematic Reviews and Meta-analyses (PRISMA), we conducted a systematic review of studies that assessed COVID-19 related cardiac abnormalities using cardiovascular magnetic resonance imaging. A total of 21 cross-sectional, case-control, and cohort studies were included in the analyses.

**Results:**

Ten studies reported CMR results <3 months after SARS-CoV-2 infection and 11 studies >3 months after SARS-CoV-2 infection. Abnormal T1, abnormal T2, elevated extracellular volume, late gadolinium enhancement and myocarditis was reported less frequently in the >3-month studies. Eight studies reported an association between biomarkers and CMR findings. Elevated troponin was associated with CMR pathology in 5/6 studies, C-reactive protein in 3/5 studies, N-terminal pro-brain natriuretic peptide in 1/2 studies, and lactate dehydrogenase and D-dimer in a single study. The rate of myocarditis via CMR was 18% (154/868) across all studies. Most SARS-CoV-2 associated CMR abnormalities resolved over time.

**Conclusions:**

There were CMR abnormalities associated with SARS-CoV-2 infection and most abnormalities resolved over time. A panel of cardiac injury and inflammatory biomarkers could be useful in identifying patients who are likely to present with abnormal CMR pathology after COVID-19. Multiple mechanisms are likely responsible for COVID-19 induced cardiac abnormalities.

## 1. Introduction

Cardiac involvement is one of the most common acute complications of SARS-CoV-2 infection [1, 2, 3] and some survivors continue to experience persistent COVID-19 related cardiac abnormalities weeks to months after recovery from acute SARS-CoV-2 infection [4]. COVID-19 survivors have an increased risk of cardiovascular morbidity and mortality along with a diverse set of COVID-19-induced cardiac complications including atrial fibrillation, heart failure, ventricular arrhythmias, pericarditis, and cardiac arrest [5]. Cardiac involvement has also been reported in non-hospitalized and mildly symptomatic COVID-19 patients [5, 6].

To date, little is certain about the mechanism of cardiac injury in COVID-19 patients [7]. SARS-CoV-2 could directly cause cardiac complications [2, 8, 9, 10, 11] because heart muscle has high density of angiotensin-converting enzyme 2 (ACE2) receptors through which SARS-CoV-2 virus enters cells [12]. Indirect effects such as systemic hypoxia, acute respiratory distress, hypercoagulation, hypotension, shock, sepsis, inflammation, cytokine storm, and other host-immune responses from COVID-19 complications could also contribute to cardiac injury [2, 8, 9, 10, 11].

Despite recent studies showing the promising role of echocardiography in predicting cardiac tissue abnormality [13], cardiac magnetic resonance (CMR) imaging remains the gold standard for detailed, non-invasive analysis of myocardial structure, function, and tissue composition, providing information regarding myocardial edema, inflammation, and fibrosis. Furthermore, CMR remains a major non-invasive diagnostic tool to help identify clinically suspected myocarditis while distinguishing ischemic from non-ischemic patterns of myocardial injury [14].

Although elevated cardiac biomarkers during the acute SARS-CoV-2 infection have been associated with COVID-19 disease severity and mortality [15, 16, 17], the use of cardiac biomarkers to predict long-term COVID-19 cardiac sequelae remains unclear [18] and warrants further investigation. These biomarkers can also be useful in identifying patients for CMR analysis.

To better understand the progression, mechanism of injury, and predictive biomarkers of COVID-19-associated cardiac sequelae, we conducted a systematic review of the CMR literature analyzing temporal COVID-19-associated cardiac manifestations.

## 2. Methods

### 2.1 Eligibility Criteria and Evidence Search

**Fig. 1.**
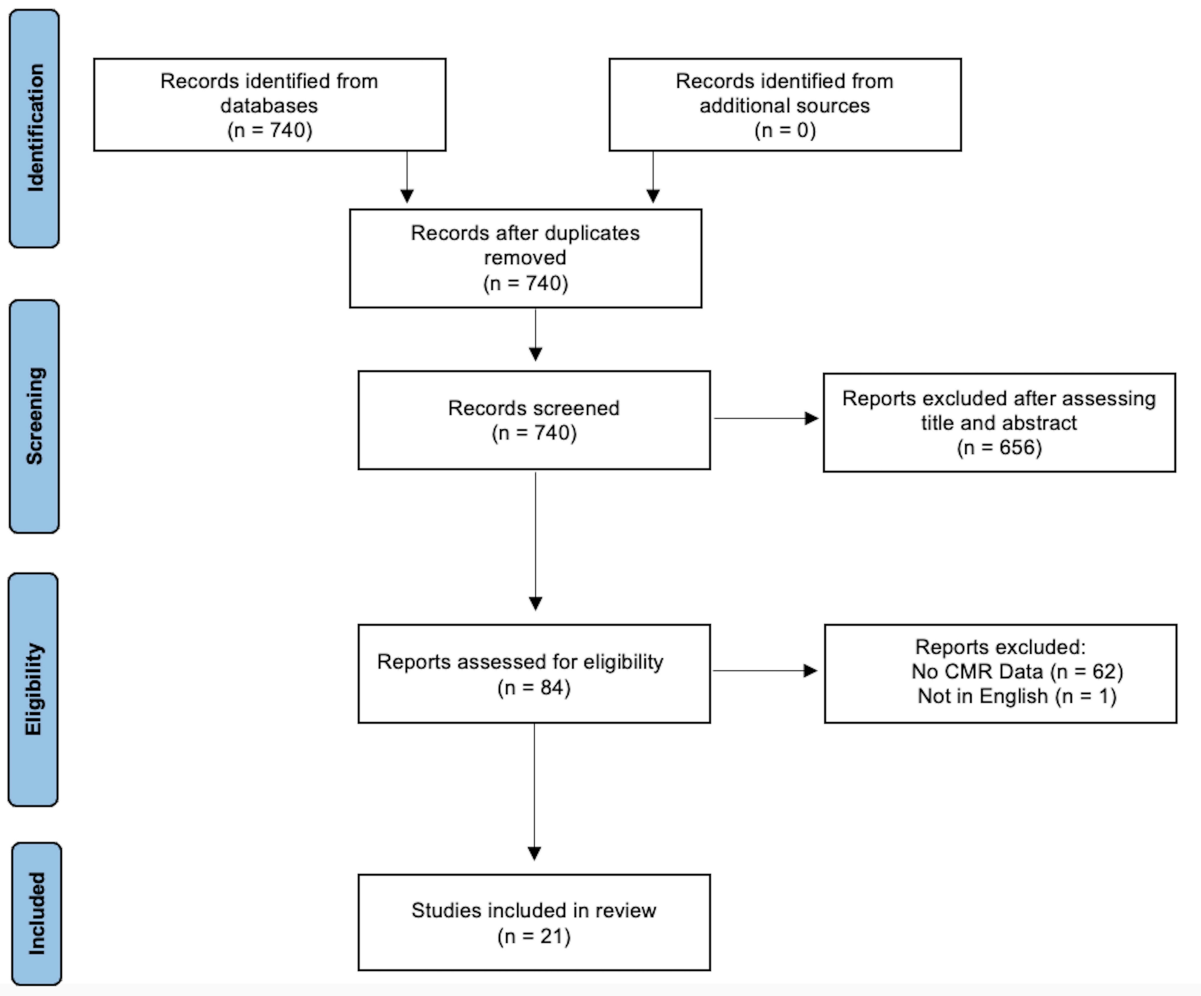
**PRISMA flow chart**.

Using Preferred Reporting Items for Systematic Reviews and Meta-analyses (PRISMA), we conducted a systematic review of studies looking at the relationship of COVID-19 cardiac sequelae and cardiovascular magnetic resonance imaging (CMR) (Fig. 1). Cross-sectional, case-control, and cohort studies were included in the analyses.

A PubMed database search from January 1, 2021 to February 23, 2022 was performed. Observational studies, including cross-sectional, case-control, and cohort studies that examined post-COVID-19 cardiac symptomatology with CMR were included in the analyses. Studies that were excluded included: (1) case reports, case series, and conference abstracts; (2) papers not written in English; (3) protocol papers, letters to the editor, and healthcare provider surveys without data; and (4) papers that did not use CMR as a data metric. Search terms, PRISMA checklists and additional information regarding the search strategy can be found in the **Supplementary Materials**. This protocol was not registered online.

### 2.2 Data Analysis

Study characteristics, including study type, origin, follow-up period and qualitative findings were systematically categorized and MRI findings, including T1, T2, late gadolinium enhancement (LGE) assessments, and the presence or absence of myocarditis, were described.

## 3. Results 

### 3.1 Study Characteristics 

Twenty-one studies fulfilled the inclusion criteria (Table 1, Ref. [19, 20, 21, 22, 23, 24, 25, 26, 27, 28, 29, 30, 31, 32, 33, 34, 35, 36, 37, 38, 39]). Seventeen were prospective, 3 were retrospective, and 1 cross-sectional. Three studies [24, 25, 30] were performed in North America, 7 in Asia [19, 23, 27, 31, 34, 37, 39] and 11 in Europe [20, 21, 22, 26, 28, 29, 32, 33, 35, 36, 38]. Male predominance was present in 10 studies [19, 21, 24, 27, 28, 31, 32, 35, 36, 38], women were more represented in 9 [20, 22, 23, 25, 26, 29, 33, 37, 39] and in 2 studies gender was not available [30, 34]. The main CMR characteristics are summarized in Table 2 (Ref. [19, 20, 21, 22, 23, 24, 25, 26, 27, 28, 29, 30, 31, 32, 33, 34, 35, 36, 37, 38, 39]).

**Table 1.** **Paper Characteristics**.

Authors	Study type	Country	N	Age (years)	Male (%)	Follow-up time	Main findings
Altay (2021) [19]	Retrospective	Turkey	15	Mean (range) 38 (30–45)	53	Median (IQR) 81 (61–105) days	LGE consistent with myocarditis was present in 46% of patients, though functional loss was not observed.
Breitbart (2021) [20]	Prospective	Germany	56	Mean (SD) 46 (12)	46	Mean (SD) 71 (66) days	Persistent LGE was found in 12% of patients, and myocarditis in 2%.
Cassar (2021) [21]	Prospective	England	58	Mean (SD) 55 (13)	59	Median (IQR) First at 2.3 months (2.1–2.5), second at 6 months (6.0–6.8)	Significant improvement in CMR parameters noted at second follow-up compared to first. CMR abnormalities were still present at second follow-up relative to controls.
Fijałkowska (2021) [22]	Prospective	Poland	95	Mean (SD) 42 (10)	23	Median (IQR) 72 (22–126) days	Cross-sectional findings of healthcare workers show persistent CMR abnormalities. Patients were largely asymptomatic.
Fu (2021) [23]	Prospective	China	34	N/A	34	6 months	CMR abnormalities were present in 65% of patients at follow-up, but specific CMR parameters were mot mentioned.
Galea (2021) [24]	Retrospective	US	27	Mean (SD) 54 (12)	70	Median (IQR) 20 (13.5–31.5) days	CMR abnormalities present in 74% of patients, including patterns consistent with myocarditis, pericarditis, and myocardial infarction.
Hanneman (2022) [25]	Prospective	Canada	47	Mean (SD) 43 (13)	49	Mean (SD) 67 (16) days	Broad improvement of CMR abnormalities, particularly myocardial inflammation indicators, was noted at follow-up.
Joy (2021) [26]	Prospective	England	731	Median (IQR) 37 (18–63)	42	Median (IQR) 6 months 9 days (5 months 26 days–6 months 20 days)	No detectable cardiovascular abnormalities after six months post-mild infection when compared with matched case control subjects.
Karaaslan (2021) [27]	Retrospective	Turkey	64	Mean (SD) 41 (14)	64	Median (IQR) 71 (17–209) days	CMR abnormalities detected in 71% of patients regardless of pre-existing conditions, though patients were largely asymptomatic and had normal troponin levels.
Kotecha (2021) [28]	Prospective	England	68	Mean (SD) 64 (12)	70	Median (IQR) 45 (30–76) days	CMR abnormalities detected in approximately 50% patients: non-infarct, myocarditis-pattern injury (27%), ischemic pathology (22%), and non-ischemic non-specific scar (5%).
Kravchenko (2021) [29]	Prospective	Finland	41	Mean (SD) 39 (13)	44	Median (IQR) 103 (88–158) days	No signs of CMR abnormalities in COVID-19 patients compared to controls at follow-up.
D. Li (2022) [30]	Prospective	US	330	19	N/A	Median (IQR) 102 (85–150) days	MIS group had greater CMR abnormalities than the non-MIS group. Persistent CMR abnormalities were noted at follow-up in both groups, though improvement was also noted.
X. Li (2021) [31]	Prospective	China	40	Mean (SD) 54 (12)	64	Mean (SD) 158 (18) days	Persistent CMR abnormalities detected at follow-up. Lack of concurrent symptoms suggests these findings are largely subclinical.
Myhre (2021) [32]	Prospective	Norway	58	Median (IQR) 56 (50–70)	56	Median (IQR) 175 (105–217) days	CMR abnormalities detected in 21% of patients at follow-up. However, resolution of CMR abnormalities over time is noted.
Petersen (2022) [33]	Prospective	Germany	443	Median (IQR) 55 (51–60)	47	Median 9.6 months	All CMR parameters between COVID-19 patients and controls were similar with the exception of T1 which was elevated in COVID-19 patients at follow-up.
Sarıçam (2021) [34]	Cross-sectional	Turkey	15	Range 20–50	N/A	Range 3 weeks–2 months	No CMR abnormalities were detected at follow-up, however, inflammation was detected via 18F-FDG uptake on cardiac PET.
Thornton (2021) [35]	Prospective	England	90	Median (IQR) 64 (54–71)	83	Median (IQR) 61 (29–146) days	At follow-up, 56% patients had evidence of myocardial scar: 17% patients had an infarct pattern LGE, 34% had a non-infarct pattern LGE, 4.4% patients had a dual/mixed pattern LGE.
Ulloa (2021) [36]	Prospective	Spain	57	Mean (SD) 59 (15)	81	Mean (SD) 81 (27) days	T2 was significantly elevated in COVID-19 patients compared to controls. Patients analyzed at <8 weeks showed more evidence of myocardial injury compared to patients analyzed at >8 weeks
Wang (2021) [37]	Prospective	China	47	Mean (SD) 47.6 (13.3)	43	Mean (SD) 102.5 (20.6) days	LGE-defined myocardial injury was present in 30% of COVID-19 patients at follow-up.
Weckbach (2021) [38]	Prospective	Germany	18	Median (IQR) 70.0 (63.5–76.5)	89	Median (IQR) 14.0 (6.8–26.8) days	At follow-up, 89% of patients showed a CMR abnormality, and 39% showed evidence of myocarditis.
Wu (2021) [39]	Prospective	China	27	Median (IQR) 63 (58–70)	30	Median (IQR) 188 (182–210 days)	LGE, but not other CMR parameters, was detected in 29.6% of patients at follow-up, suggesting persistent fibrosis 6 months post-COVID-19.

**Table 2.** **Cardiac MRI Findings**.

Paper		Increased T1	Increased T2	Increased ECV	Presence of LGE	Myocarditis
During Hospitalization (375 patients)						
	Weckback [38]	Yes	Yes	Yes	Yes	Yes
	Galea [24]	Yes	Yes	Yes	Yes	Yes
	D. Li [30]	-	-	-	-	Yes
3 weeks-3 Months (565 patients)						
	Saricam [34]	-	-	-	No	No
	Thornton [35]	-	-	-	Yes	Yes
	Hanneman [25]	Yes	Yes	Yes	Yes	-
	Kotecha [28]	Yes	Yes	-	Yes	Yes
	Breitbart [20]	-	-	-	Yes	Yes
	Karaaslan [27]	-	-	-	Yes	-
	Fijalkowska [22]	Yes	Yes	Yes	Yes	Yes
	Cassar [21]	Yes	No	No	Yes	Yes
	Altay [19]	No	No	No	Yes	Yes
	Ulloa [36]	No	Yes	No	Yes	No
>3 Months (1566 patients)						
	D. Li [30]	Yes	Yes	-	Yes	-
	Kravchenko [29]	No	No	No	No	No
	Wang [37]	No	-	-	Yes	-
	Hanneman [25]	No	No	No	-	-
	X. Li [31]	Yes	-	Yes	-	Yes
	Myhre [32]	No	No	No	No	No
	Cassar [21]	No	No	No	No	Yes
	Fu [23]	-	-	-	-	-
	Joy [26]	No	No	No	No	No
	Wu [39]	No	-	No	Yes	-
	Peterson [33]	Yes	No	-	No	-

“Yes” refers to presence of the CMR finding; “No” refers to absence of the CMR finding; “-“ refers to an unreported CMR findings.

Three studies reported [24, 30, 38] CMR results during the acute COVID-19 hospitalization. The mean time from COVID-19 diagnosis to follow-up was 20 days, representing 375 patients. In all three studies at least one sign of myocardial injury and myocarditis was noted. Only two out of the three studies reported T1, T2, extracellular volume (ECV), and LGE data, with elevations of T1, T2 and ECV in addition to LGE present in both studies.

Ten studies [19, 20, 21, 22, 25, 27, 28, 34, 35, 36] reported CMR results three weeks to three months after SARS-CoV-2 infection, encompassing a total of 565 patients. Abnormal T1 values were noted in 3/6 studies, abnormal T2 values in 3/6 studies, elevated ECV in 2/5 studies, LGE in 9/10 studies, and myocarditis in 6/8 studies.

Eleven studies [21, 23, 25, 26, 29, 30, 31, 32, 33, 37, 39] reported CMR results beyond three months after SARS-CoV-2 infection. The average follow-up time was 15 weeks to 9.6 months with a total of 1566 patients represented. Abnormal T1 was reported in 3/10 studies, abnormal T2 in 1/7 studies, elevated ECV in 1/7 studies, LGE in 3/8 studies, and myocarditis in 1/4 studies.

Tabulating available data, we found the rate of myocarditis via CMR to be 18% (154/868) across all studies.

### 3.2 Laboratory Blood Biomarkers

Cardiac and inflammatory biomarkers were reported in 18 out of the 21 studies, the most common of which include troponin, N-terminal pro b-type natriuretic peptide (NT-proBNP), and C-reactive protein (CRP). Additional biomarkers reported included IL-6, D-dimer, LDH, creatinine kinase (CK), procalcitonin (PCT), myoglobin (MYO), ferritin, and myeloperoxidase.

A direct association between these biomarkers and CMR findings at either hospital admission or follow-up was reported in 8 of the 18 studies (Table 3, Ref. [19, 23, 24, 27, 30, 32, 37, 39]). In these studies, an elevated troponin was associated with CMR pathology in 5/6 studies, CRP in 3/5 studies, NT-proBNP in 1/2 studies, and LDH and D-dimer in a single study. Although the remaining 10 studies did measure biomarkers, they were not included in the analysis because they did not analyze them in relation to CMR pathology.

**Table 3.** **Biomarkers associated with CMR-defined cardiac injury**.

Paper	Main findings	Biomarkers measured at follow-up (F) or admission (A)	Biomarkers associated with cardiac injury	Biomarkers not associated with cardiac injury
Galea [24]	Increase T2 correlated with hs-cTnT and ECV measured at follow-up.	F	Troponin, CRP, D-dimer	-
D. Li [30]	Admission CRP elevation not correlated with CMR findings at follow-up.	A	-	CRP
Altay [19]	In-hospital hs-cTnI correlated with LGE at follow-up. Low correlation between CRP and LGE.	A	Troponin, CRP	
Karaaslan [27]	CRP elevated in those with LGE at follow-up. Hs-TnT and NT-proBNP were not.	F	CRP	hs-TnT, NT-proBNP
Wang [37]	Significant admission TnI elevation in LGE group vs non-LGE group	A	Troponin	CK, CKMB, MYO, CRP
Myhre [32]	Admission cTnT and NT-proBNP associated with CMR pathology at follow-up.	A	Troponin, NT-proBNP	IL-6, CRP, PCT
	Inflammatory markers (IL-6, CRP, PCT) were similar in those with and without CMR pathology at FU.			
Fu [23]	Elevated LDH at admission associated with RV dysfunction and LV dysfunction at follow-up.	A	LDH	-
Wu [39]	LGE at follow-up was more common in those with cardiac injury (defined as hs-cTnI ≥99%) at admission.	A	Troponin	-

## 4. Discussion

### 4.1 Time-Wise Analysis of CMR Cardiac Sequala

In this systematic review, we found that CMR abnormalities associated with SARS-CoV-2 infection resolved over time. T1 (indicative of myocardial fibrosis and/or edema) and T2 (specific for myocardial edema [40]) values, in addition to ECV, were elevated in the acute COVID-19 hospital setting. Compared to the acute-disease phase, the 0–3 months phase showed a decrease in T1 and T2 elevations consistent with a decline in myocardial edema, and a decreased ECV elevation compatible with decreased myocardial inflammation. Compared to the 3 week–3-month phase, fewer studies demonstrate elevated T1, T2, and ECV in the >3-month phase, further suggesting that myocardial edema and inflammation of COVID-19 infection resolves over time. The persistence of LGE in the 3 week to 3 month phase might be consistent with myocardial scar formation and regional myocardial fibrosis. However, the decrease in LGE in the >3-month studies suggests that in addition to myocardial fibrosis, LGE is detecting reversible myocardial injury, consistent with studies of non-COVID-19 myocarditis and with some cases of myocardial infarction [41]. Likewise, CMR-defined myocarditis also showed timewise resolution.

The hypothesis that CMR abnormalities resolve over time is supported by the four prospective studies within the overall cohort that analyzed COVID-19 patients at two different follow-up times [21, 25, 30, 36]. Cassar *et al*. [21] found evidence of decreased T1, ECV, and LGE at a 6-month follow-up compared to an earlier 2–3-month follow-up of the same patients. Leslie Li *et al*. [30] found that 75% of patients showed complete resolution of myocardial edema/inflammation at a 138-day follow-up relative to a 36-day follow-up, as well as a significant improvement of LGE burden over that same time. Hanneman *et al*. [25] showed increased LVEF, decreased T1, T2, and ECV at a 119-day follow-up compared to a 67-day follow-up in those with FDG uptake. Using a different analysis, Ulloa *et al*. [36] reported that patients analyzed via CMR at <8 weeks from COVID-19 diagnosis had significantly less circumferential and radial strain compared to those analyzed at >8 weeks. Together, there is strong evidence in support of the hypothesis that cardiac abnormalities found on CMR resolve over time. Whether the persistence or resolution of CMR findings offer prognostic value for long-term adverse cardiovascular events remains to be determined.

### 4.2 Biomarkers and Risk Factors of Cardiac Involvement

Quantitative analysis of a panel of biomarkers may be useful in predicting patients who are more likely to have COVID-19-associated CMR abnormalities and, thus, who should be referred for CMR analysis. Myhre *et al*. [32] showed that elevated levels of NT-proBNP and troponin on admission were associated with positive cardiac MRI findings during long-term follow-up, but inflammatory markers were not. Galea *et al*. [24] report that post-acute COVID-19 patients with elevated troponin-T values have higher T2 and ECV values than patients below the upper limit troponin cutoff with a sensitivity of 92.9% and specificity of 76.9%. Multiple authors have reported an association between CMR abnormalities and admission troponin I levels, even in the absence of clinical symptoms [19, 27, 37, 39], including the presence of LGE many months after infection. This suggests that patients with elevated troponin-I levels at admission should be monitored closely for many months after discharge.

Although a meta-analysis by Fu *et al*. [42] supports the association of severity of the acute illness phase of COVID-19 and biomarker-defined cardiac injury (a seven-fold higher prevalence of biomarker-defined cardiac injury in severe compared to non-severe patients), others reported that disease severity during the acute illness is not associated with CMR pathologic findings [24, 27, 32]. This dichotomy may be explained by the fact that biomarkers such as troponin-I can be elevated in non-cardiac injuries, including renal insufficiency and pulmonary embolism, which are also a result of COVID-19.

Multiple risk factors, such as acute COVID-19 illness severity, older age, male sex, preexisting cardiovascular disease, hypertension, and COPD have been previously suggested to be associated with higher rates of myocardial injury as defined by elevated biomarkers [43, 44]. In our review, some studies found evidence in support of an association with older age [23, 32, 35] while others did not [27]. This may be due, in part, to the higher disease severity of COVID-19 seen in those studies that show an association [23, 32, 35]. Furthermore, it is unknown whether the CMR changes seen in older patients are preexisting rather than COVID-19 related. Similarly, the relationship between male sex and CMR evidence of injury is unclear [27, 32, 35, 45, 46]. In a pooled meta-analysis of 4 studies, Zou *et al*. [43] found no statistical significance between male sex to the appearance of cardiac injury.

### 4.3 Mechanism of Cardiac Injury

Multiple mechanisms of cardiac injury due to SARS-CoV-2 infection have been postulated. These include ischemic and non-ischemic pathways [47]. Ischemic mechanisms, which include acute coronary artery and arteriole occlusion, involve pathological endothelial activation and thrombosis. Non-ischemic mechanisms include myocarditis, systemic hyperinflammation, hypoxic injury due to severe respiratory infection, and down regulation of ACE2 receptors. All nine studies which separated cardiac injury into non-ischemic and ischemic causes reported a greater prevalence of non-ischemic injury [20, 22, 24, 28, 29, 32, 35, 36, 38] suggesting that thrombosis and endothelial damage driven cardiac injury is less likely in COVID-19 associated cardiac injury. While Thornton *et al*. [35] reported the prevalence ischemic injury in their cohort to be 17%, they concluded that this most likely reflected pre-existing comorbidities. Interestingly, Saricam *et al*. [34] report that COVID-19 patients with markers of cardiac injury have higher nitric oxide levels than those with no markers. Given that nitric oxide is a potent anti-inflammatory molecule and vasodilator released by endothelial cells, this suggests that endothelial dysfunction and a subsequent prothrombotic state may be a mechanism of COVID-19 cardiac injury. Though it should be noted that this is a relatively minor finding, and that the majority of papers in this review reported a mainly non-ischemic mechanism of injury.

Cytokine storm in severe COVID-19 results in multiorgan damage including the lung, heart, kidney, testis, and liver [48, 49, 50, 51, 52, 53, 54] due to vascular hyperpermeability, edema, and hypercoagulation, has been shown to be accompanied by elevation of the inflammatory markers IL-6, IL-8, and CRP in those with cardiac injury [55, 56, 57]. Normalization of these biomarkers over time is accompanied by a trend towards normalization of CMR abnormalities [25]. Moreover, it has been demonstrated that COVID-19 patients with multisystem inflammatory syndrome (MIS), characterized by a systemic hyperinflammatory state, have significantly elevated native T1, whereas only regional inflammation and edema was noted in the non-MIS group [30]. Likewise, Cassar *et al*. [21] and Ulloa *et al*. [36] also conclude that CMR abnormalities are likely the result of cytokine-mediated injury.

Myocarditis is another mechanism of cardiac injury in COVID-19 patients. Given the patient populations analyzed in these studies, the 18% (154/868) incidence we report is likely an overestimation of the true prevalence of myocarditis in COVID-19 infected patients. This means that although myocarditis may be a mechanism of cardiac injury in COVID-19 patients, it is not the only mechanism of injury. This conclusion is further supported by endomyocardial biopsies which, in addition to confirming myocarditis through the presence of lymphocytic infiltrate with associated myocyte necrosis, have also confirmed macrophage-dominated inflammation without any myocyte necrosis, which is inconsistent with myocarditis-mediated cardiac injury [38, 58].

### 4.4 Limitations

The studies included in this systematic review are heterogeneous in nature. Variability in the study design, size, use of controls, CMR parameters, and patient populations resulted in patient selection bias. Many studies did not include proper control groups and thus the incidence of cardiac abnormalities could not be compared with their natural incidence. Due to the heterogenous nature of the studies, it was impossible to report more granular detail beyond the presence and absence of CMR abnormalities. Some CMR findings could be pre-existing and were not caused or associated with SARS-CoV-2. The notion that these CMR findings were observed were at least likely exacerbated by SARS-CoV-2 infection. Arrythmias, which are a reported complication of COVID-19, could not be analyzed [59, 60]. Despite COVID-19 being a systemic disease, mechanisms of cardiac injury were only analyzed in the context of the cardiovascular system. Lastly, there could be unintentional reporting biases in published literature as many studies were retrospective studies and case series reports.

## 5. Conclusions

SARS-CoV-2 infection is associated with a wide range of CMR pathology and there is evidence that SARS-CoV-2 associated CMR pathology largely resolves over time. A cardiac injury and inflammatory biomarker panel may be useful in identifying patients who are likely to experience persistent COVID-19 CMR pathology. Such a biomarker panel may be used to inform the use of CMR in patients post-COVID-19. There are likely multiple mechanisms by which COVID-19 induces cardiac injury. The significance of subclinical CMR findings with respect to long-term outcomes remains to be determined. Longer term follow-up CMR studies are needed.

## Supplementary Material

Supplementary material associated with this article can be found, in the online version, at https://doi.org/10.31083/j.rcm2312389.
