# Supplementary material for: Cardiac Magnetic Resonance Imaging of COVID-19-Associated Cardiac Sequelae: A Systematic Review
Source: Rev Cardiovasc Med. 2022 Nov 30;23(12):389. doi: 10.31083/j.rcm2312389 (PMC11270474; doi:10.31083/j.rcm2312389)
Supplement: Supplementary file 1 [file 2153-8174-23-12-389-s1.docx]

**PUBMED Search Strategy:**

((COVID*[Title/Abstract]) OR (SARS-CoV-2[Title/Abstract]) OR coronavirus[Title/Abstract])

AND

((Card*[Title/Abstract]) OR (heart[Title/Abstract]) OR (myocard*[Title/Abstract])

AND

((follow*[Title/Abstract]) OR (month*[Title/Abstract]) OR (year*[Title/Abstract]) OR (long*[Title/Abstract]) OR (recover*[Title/Abstract]) OR (sequelae[Title/Abstract]))

AND

(echo*) OR (CMR) OR (magnetic)
